# Supplementary material for: Neural circuits underlying context-dependent competition between defensive actions in Drosophila larvae
Source: Nat Commun. 2025 Jan 28;16:1120. doi: 10.1038/s41467-025-56185-2 (PMC11775277; doi:10.1038/s41467-025-56185-2)
Supplement: Supplementary file 1 — Supplementary Information [file 41467_2025_56185_MOESM1_ESM.pdf]

Supplementary Information

Neural circuits underlying context-dependent competition between defensive actions in *Drosophila* larvae

Maxime Lehman[1], Chloé Barré[#,2,3], Md Amit Hasan[#,1], Benjamin Flament[1], Sandra Autran[1], Neena Dhiman[4,5], Peter Soba[4,5], Jean-Baptiste Masson[2,3], Tihana Jovanic[1]*

[#]These authors contributed equally

[1]Université Paris-Saclay, CNRS, Institut des neurosciences Paris-Saclay, 91400, Saclay, France.

[2]Institut Pasteur, Université Paris Cité, CNRS UMR 3571, Decision and Bayesian Computation, 75015 Paris, France.

[3]Epiméthée, INRIA, 75013 Paris, France.

[4]Institute of Physiology and Pathophysiology, Friedrich-Alexander-Universität Erlangen- Nürnberg, 91054 Erlangen, Germany.

[5]LIMES Institute, Department of Molecular Brain Physiology and Behavior, University of Bonn, Carl-Troll-Str. 31, 53115 Bonn, Germany.

*Corresponding author: tihana.jovanic@cnrs.fr

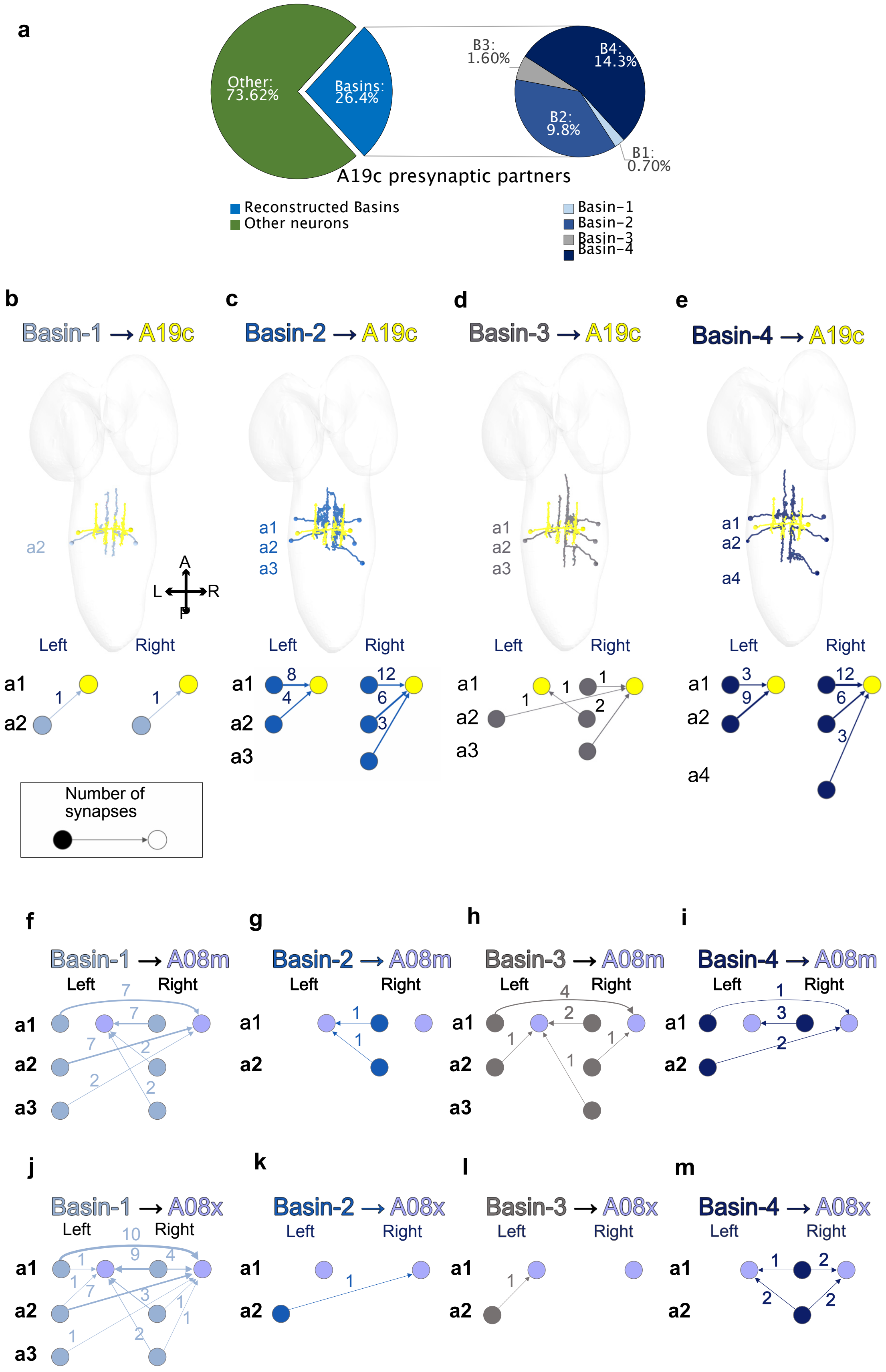

Supplementary Figure 1. a. Distribution of all A19c inputs shown as fractions of inputs of A19c from Basins and non-Basin neurons. **b-e. Top** EM reconstruction images of Basin neurons in segments and A19c neurons b-e**. Bottom** Connectivity of Basin 1-4 - A19c in neuromeres A1-4, left and right. **f-i.** Connectivity of Basins 1-4 - A08m in neuromeres A1-4, left and right. **j-m.** Connectivity of Basins 1-4 - A08x in neuromeres A1-4, left and right. The source data are provided in Source Data 1 and 2.

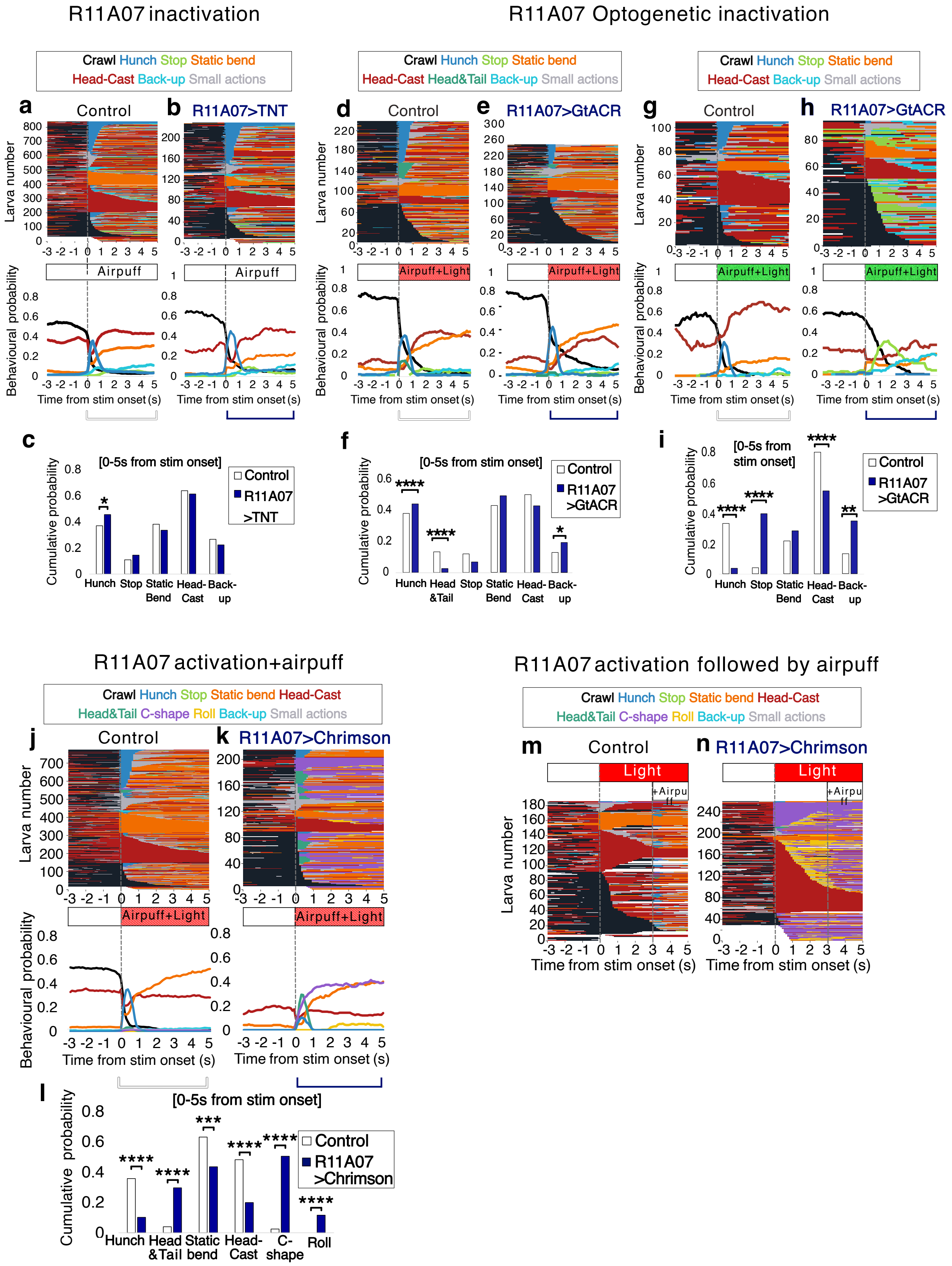

R11A07 inactivation
R11A07 Optogenetic inactivation
Crawl Hunch Stop Static bend
Head-Cast Back-up Small actions
Crawl Hunch Stop Static bend
Head-Cast Head&Tail Back-up Small actions
a
Control
b
R11A07>TNT
d
Control
e
R11A07>GtACR
g
Control
h
R11A07>GtACR
Larva number
Airpuff
Airpuff+Light
Behavioural probability
Time from stim onset (s)
c
[0-5s from stim onset]
Control
R11A07
>TNT
Cumulative probability
Hunch
Stop
Static Bend
Head-Cast
Back-up
f
R11A07
>GtACR
Head &Tail
i
[0-5s from stim onset]
R11A07 activation+airpuff
R11A07 activation followed by airpuff
Crawl Hunch Stop Static bend Head-Cast
Head&Tail C-shape Roll Back-up Small actions
j
Control
k
R11A07>Chrimson
m
Control
n
R11A07>Chrimson
Light
+Airpuff
l
[0-5s from stim onset]
R11A07
>Chrimson
Hunch
Head &Tail
Static bend
Head-Cast
C-shape
Roll

Supplementary Figure 2. a-c. TNT inactivation of R11A07 neurons during air puff responses (4 m/s) **a.** control larvae (attP2>TNT, n=818) **b.** larvae with inactivated R11A07 neuron (R11A07>TNT, n=225). **a,b.** ethogram (top) with one line corresponding to one individual and each color corresponding to a different action. Mean behavioral probability over time (bottom). Stim onset at 60 s. **c.** behavioral probability cumulated over the first five seconds after air puff onset, control larvae (white) and larvae with R11A07 inactivated (dark blue) **d-f.** Optogenetic inactivation of R11A07 with GtACR1 during air puff responses (4 m/s) using red light (2 mW/cm²). **d.** control larvae (attP2>GtACR1, n = 242), **e.** larvae with R11A07 neurons optogenetically inactivated (R11A07>GtACR1, n=307). **d,e.** ethogram (top), mean behavioral probabilities over time (bottom). Stim onset at 60 s. **f.** behavioral probability cumulated over the first five seconds after air puff onset. Control larvae (white) and larvae with R11A07 optogenetically inactivated (dark blue). **g-i.** Optogenetic inactivation of R11A07 with GtACR1 during air puff responses (4 m/s) using green light (0.9 mW/cm2). **g.** control larvae (attP2>GtACR1, n = 105), **h.** larvae with R11A07 neurons optogenetically inactivated (R11A07>GtACR1, n=95). **g,h.** ethogram (top), mean behavioral probabilities over time (bottom). Stim onset at 60 s. **i.** behavioral probability cumulated over the first five seconds after air puff onset. Control larvae (white) and larvae with R11A07 optogenetically inactivated (dark blue). **j-l.** Optogenetic activation of R11A07 neurons during air puff (4 m/s) responses. Red light (0.3mW/cm²) was used for stimulation. **j.** control larvae (attP2>CsCrimson, n=765, **k.** larvae with R11A07 neurons optogenetically activated (R11A07>CsCrimson, n=214). **j,k.** ethogram (top), mean behavioral probabilities over time (bottom). Stim onset at 60 s. **l.** behavioral probability cumulated over the first five seconds after air puff onset. Control larvae (white) and larvae with R11A07 optogenetically activated (dark blue). **m,n.** Larval responses to optogenetic activation (light at 60s) then air puff, delivered 3s after the light activation (at 63 s) **m.** control larvae (attP2>CsChrimson, n=186) **n.** larvae with activated R11A07 neurons (R11A07>-CsChrimson, n=255). For all barplots: *:p<0.05, **:p<0.005, ***:p<0.0005,****:p<0.0001, Chi² test, two-sided. The source data and p values are provided in Source Data 5.

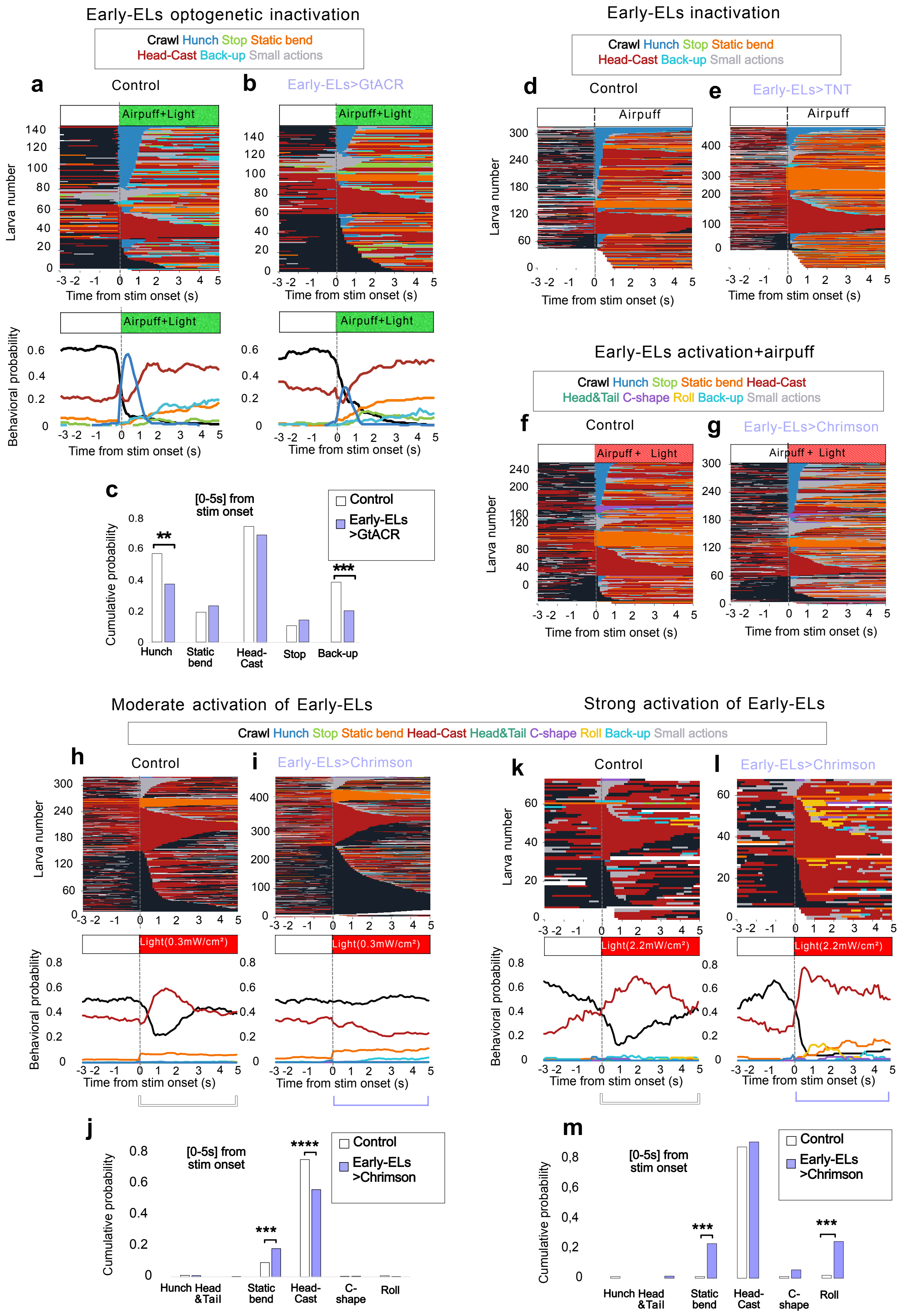
Early-ELs optogenetic inactivation
Crawl Hunch Stop Static bend
Head-Cast Back-up Small actions
a
Control
b
Early-ELs>GtACR
Airpuff+Light
Larva number
Time from stim onset (s)
Behavioral probability
c
[0-5s] from stim onset
Control
Early-ELs >GtACR
Cumulative probability
**
***
Hunch
Static bend
Head-Cast
Stop
Back-up
Early-ELs inactivation
Crawl Hunch Stop Static bend
Head-Cast Back-up Small actions
d
Control
e
Early-ELs>TNT
Airpuff
Early-ELs activation+airpuff
Crawl Hunch Stop Static bend Head-Cast
Head&Tail C-shape Roll Back-up Small actions
f
Control
g
Early-ELs>Chrimson
Airpuff + Light
Moderate activation of Early-ELs
Strong activation of Early-ELs
Crawl Hunch Stop Static bend Head-Cast Head&Tail C-shape Roll Back-up Small actions
h
Control
i
Early-ELs>Chrimson
k
Control
l
Early-ELs>Chrimson
Light(0.3mW/cm²)
Light(2.2mW/cm²)
j
[0-5s] from stim onset
Control
Early-ELs >Chrimson
****
***
Hunch Head &Tail
Static bend
Head-Cast
C-shape
Roll
m
[0-5s] from stim onset
Control
Early-ELs >Chrimson
***
***

Supplementary Figure 3. Early-born ELs inactivation and optogenetic activation experiments. **a-c.** Optogenetic inactivation of early-born ELs (EL-Gal4_R11F02GAL80>GtACR1, green light, 0.9 mW/cm^2 irradiance) during air puff (4 m/s). **a.** Control (EL-Gal4_R11F02GAL80>FCF_Attp2), n=144). **b.** eELs>GtACR1 n=151 **c.** behavioral probability cumulated over the first five seconds after light onset**.** Control larvae are in white and larvae with early-born EL neurons optogenetically inactivated are in light blue **d,e.** Inactivation of early-born ELs impacts behavioral responses to strong air puff. **d.** control (CantonS>TNT,n=254). **e.** inactivation of early-born ELs (EL-Gal4_R11F02GAL80>TNT, n=420). **f,g.** Early-born ELs optogenetic activation (EL-Gal4_R11F02GAL80>CsChrimson, red light, 0.3mW/cm^2 irradiance) during air puff (4 m/s) **f.** Control (animals reared on food medium without ATR, n=248). **g.** eELs>CsCrimson (animals reared on food medium supplemented with ATR, n=286). **h-j.** optogenetic activation of early-born eELs (EL-Gal4_R11F02GAL80>CsCrimson) at 0.3mW/cm^2 irradiance. **h.** Control (animals reared on food medium without ATR , n=303) Top: ethograms, Bottom: mean behavioral probability over time. **i.** ELs>CsChrimson (animals reared on food medium supplemented with ATR, n=465). **j.** behavioral probability cumulated over the first five seconds after light onset**. k-m.** optogenetic activation of early-born ELs at 2.2mW/cm^2 irradiance. **k.** Control (animals reared on food medium without ATR , n=62). **l.** ELs>CsCrimson (animals reared on food medium supplemented with ATR, n=60). **m.** behavioral probability cumulated over the first five seconds after light onset**.** Note that behavioral probabilities for **f,g** and **g,h** can be found in Fig. 1. For all barplots: *:$p<0.05$, **:$p<0.005$, ***:$p<0.0005$, ****:$p<0.0001$,Chi^2 test, two-sided. The source data and p values are provided in Source Data 5.

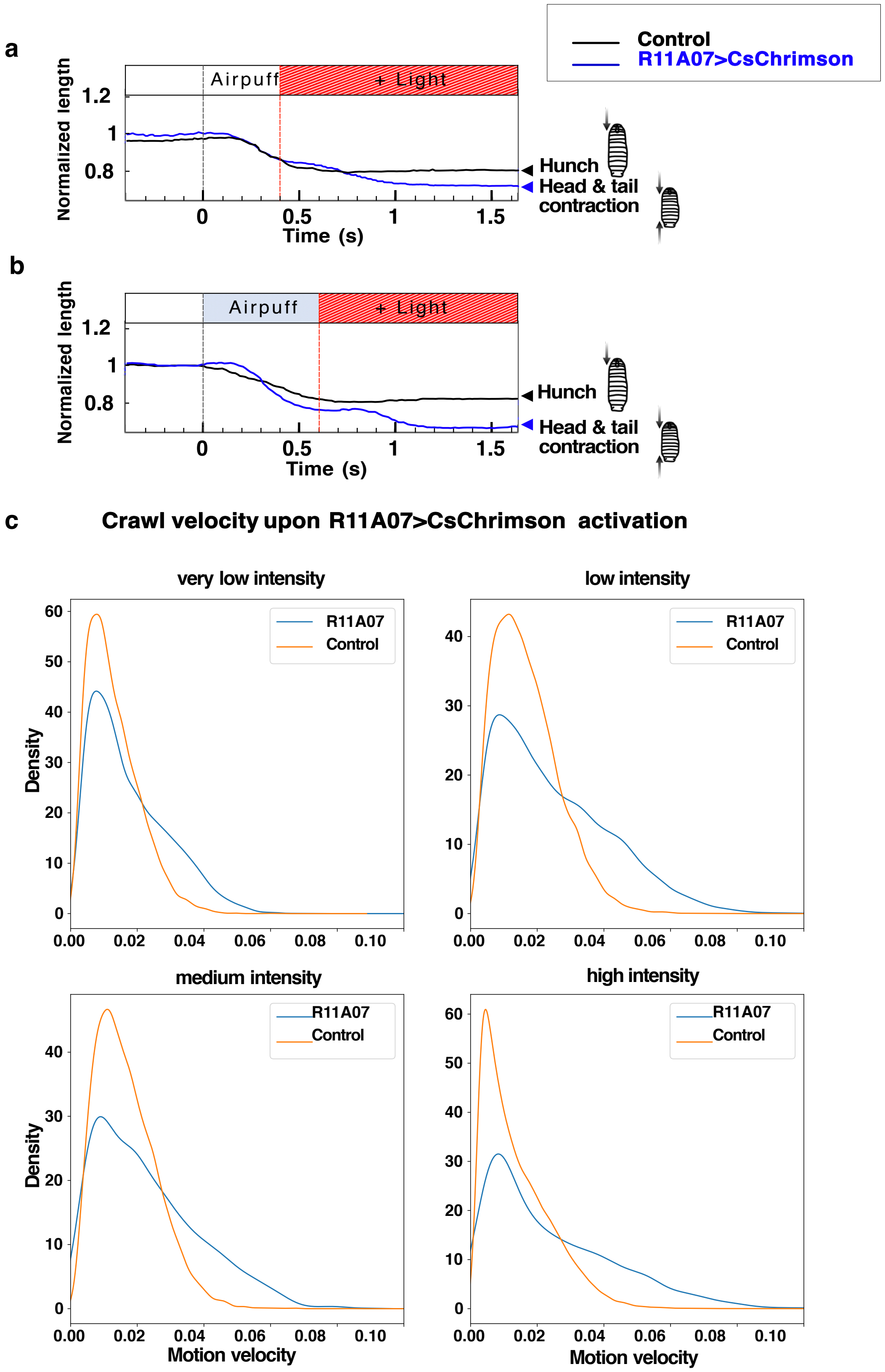

Supplementary Figure 4. Impact of R11A07 optogenetic activation on larval length and speed
a-b. Impact of delayed optogenetic activation of neurons labeled by the R11A07 driver on larval length in response to air puff. Control: attP2>CsCrimson, R11A07>CsChrimson larvae were reared on food supplemented with ATR **a.** Light delivered 0.4s after air puff onset. **b.** light delivered 0.6s after air puff onset. **c.** Distribution of motion velocities during Crawls (velocity of the center of mass expressed in normalized body lengths per second (s^{-1})) during the first 10 seconds upon optogenetic activation of R11A07 neurons compared the control. Different intensities of light are shown: very low, low (0.1 mW/cm²), medium (0.2 mW/cm²), strong (0.3, mW/cm²)

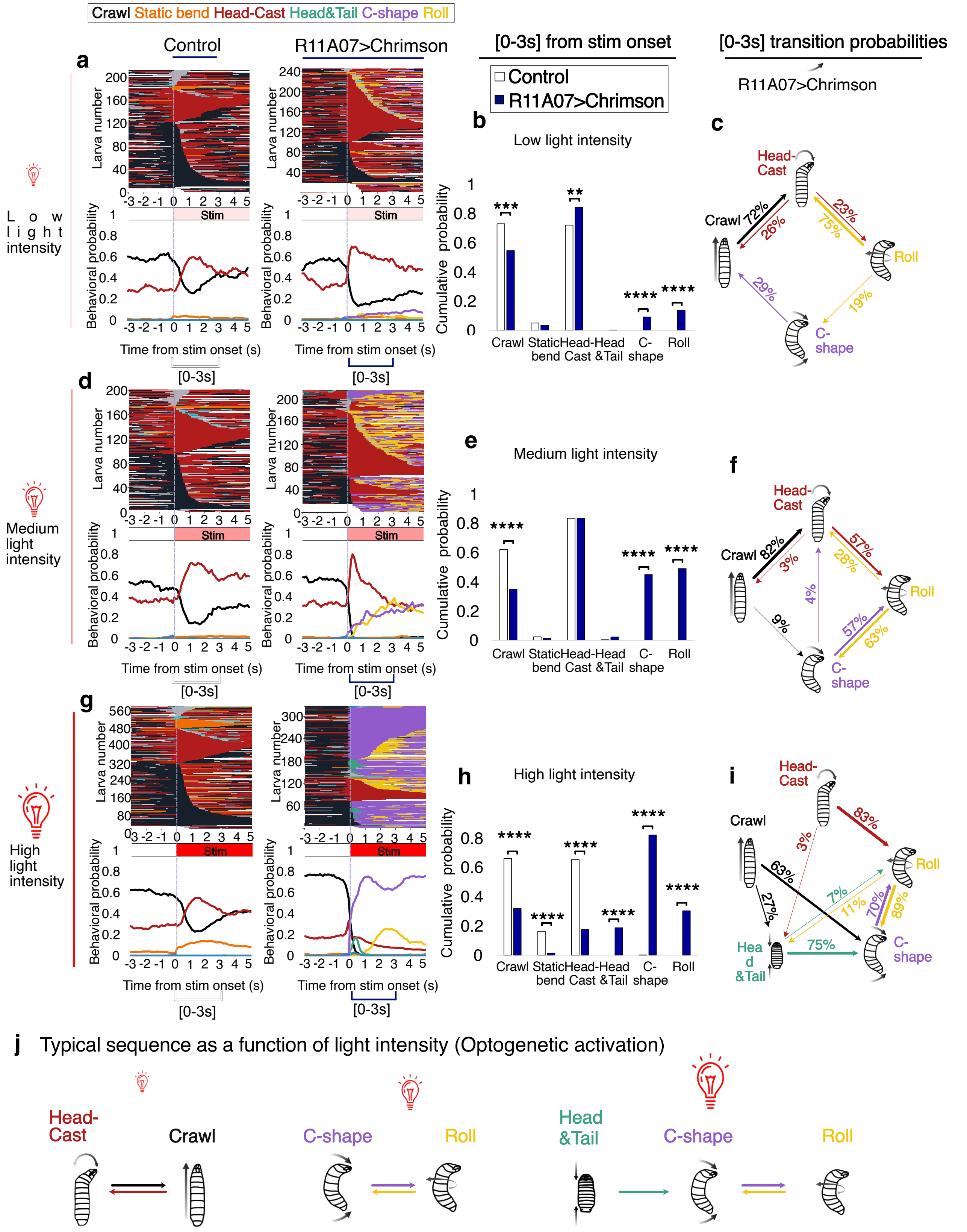

Supplementary Figure 5. Ethograms, behavioral probabilities over time, cumulative and transition probabilities for different levels of optogenetic activation of R11A07 neurons (R11A07>CsChrimson) and the control (attP2>CsChrimson) **a-c.** Low, **d-f.** Medium **g-i.** High light intensity**. j.** Representative sequences at different levels of optogenetic activation. Cumulative and transition probabilities were computed during the first 3 seconds of light stimulation. At higher light intensities more larvae transitioned from a Head Cast into a Roll (**c,f,i**). For all barplots: *:$p<0.05$, **:$p<0.005$, ***:$p<0.0005$, ****:$p<0.0001$,Chi² test, two-sided. The source data and p values are provided in Source Data 3 and 5.

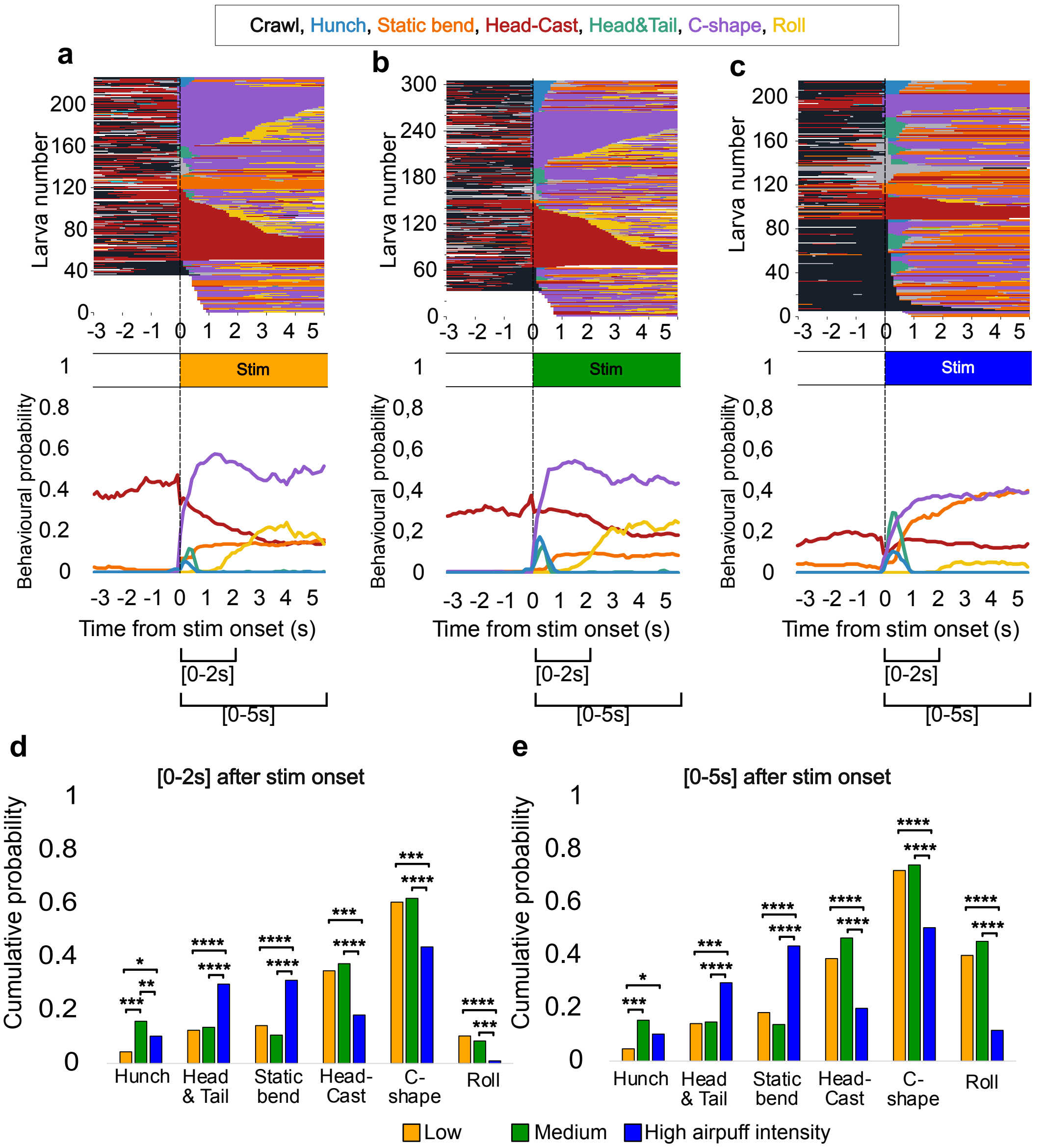

Supplementary Figure 6. Influence of air puff on escape response induced by R11A07 activation. a. behavioral probabilities in response to 0.3mW/cm² light and 2m/s air puff, **b.** behavioral probabilities in response to 0.3mW/cm² light and 3m/s air puff, **c.** behavioral probabilities in response to 0.3mW/cm² light and 4m/s air puff. **d.** behavioral probability cumulated over the first two seconds after stim onset and **e**. behavioral probability cumulated over the first five seconds after stim onset. For all barplots: *:p<0.05, **:p<0.005, ***:p<0.0005, ****:p<0.0001,Chi² test, two-sided. The source data and p values are provided in Source Data 5.

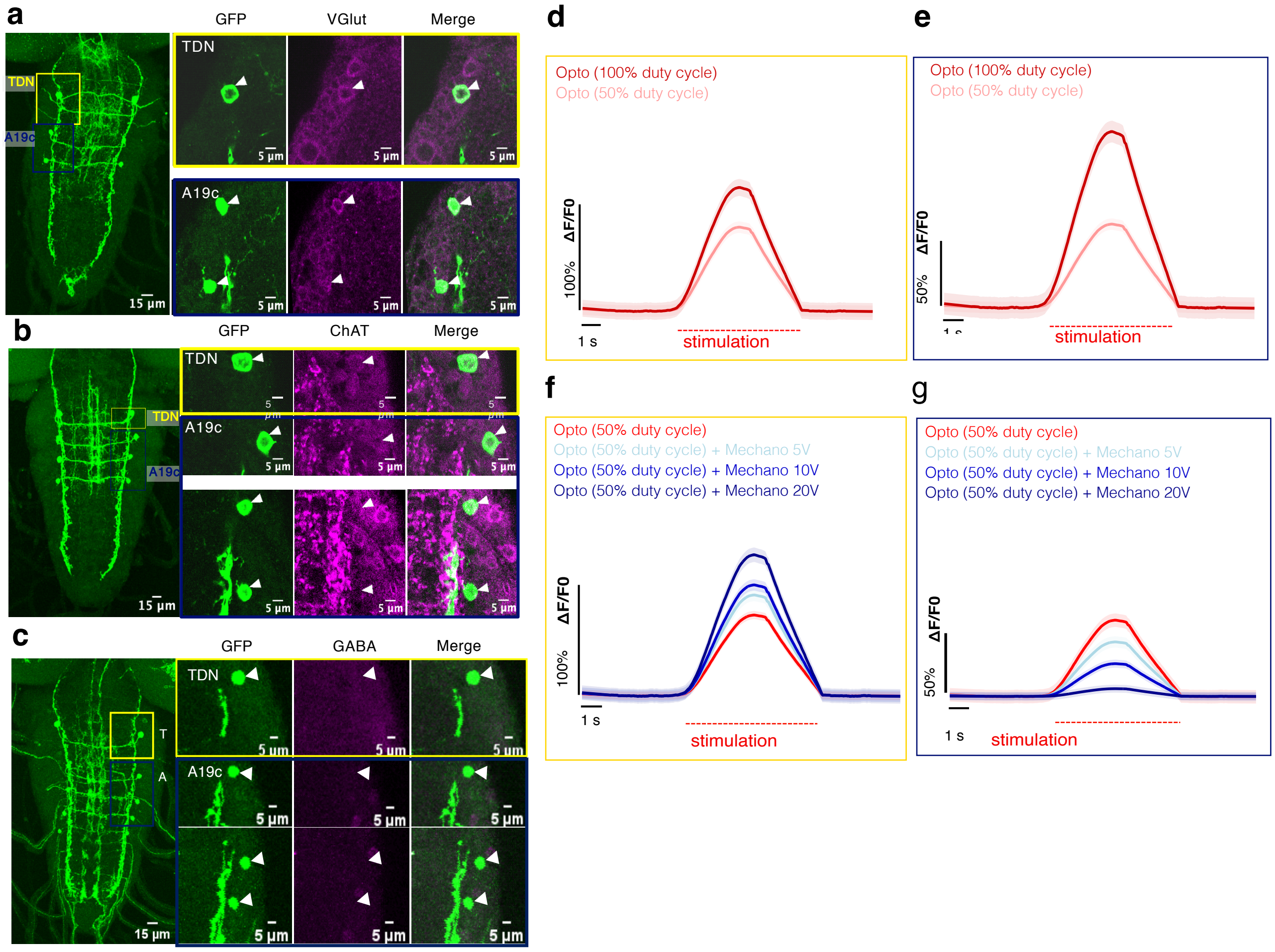

Supplementary Figure 7. a-c. Immunohistochemical labeling of TDN and A19c neurotransmitters. GFP labels the GAL4 expression patterns **a.** Glutamatergic (VGlut), n=2 **b.** Cholinergic (ChAT), n=3 **c.** GABA, n=5. Genotypes used: **a.** 65E09-AD;11A07-DBD>GFP. **b.** 11A07AD; 65E09-DBD>GFP. **c.** R11A07>GFP (image as in Fig. 5a, but only the VNC portion is shown). Magenta corresponds to neurotransmitter labeling **d,e.** Calcium imaging, using GCaMP6s, upon optogenetic activation of R11A07 neurons. **d.** TDN response (n=8 animals) **e.** A19c response (n= 8 animals). Light stimulation lasted 5s. **f,g.** Calcium imaging, using GCaMP6s, upon optogenetic activation of R11A07 neurons and different levels of mechanical stimulation. **f.** TDN response (n= 8 animals). **g.** A19c response (n= 8 animals). Light and mechanical stimulation lasted 5s. Mean and s.e.m are shown. In whole VNC images scale bar is 15 µm, in zoomed-in images: scale bar is 5 µm.

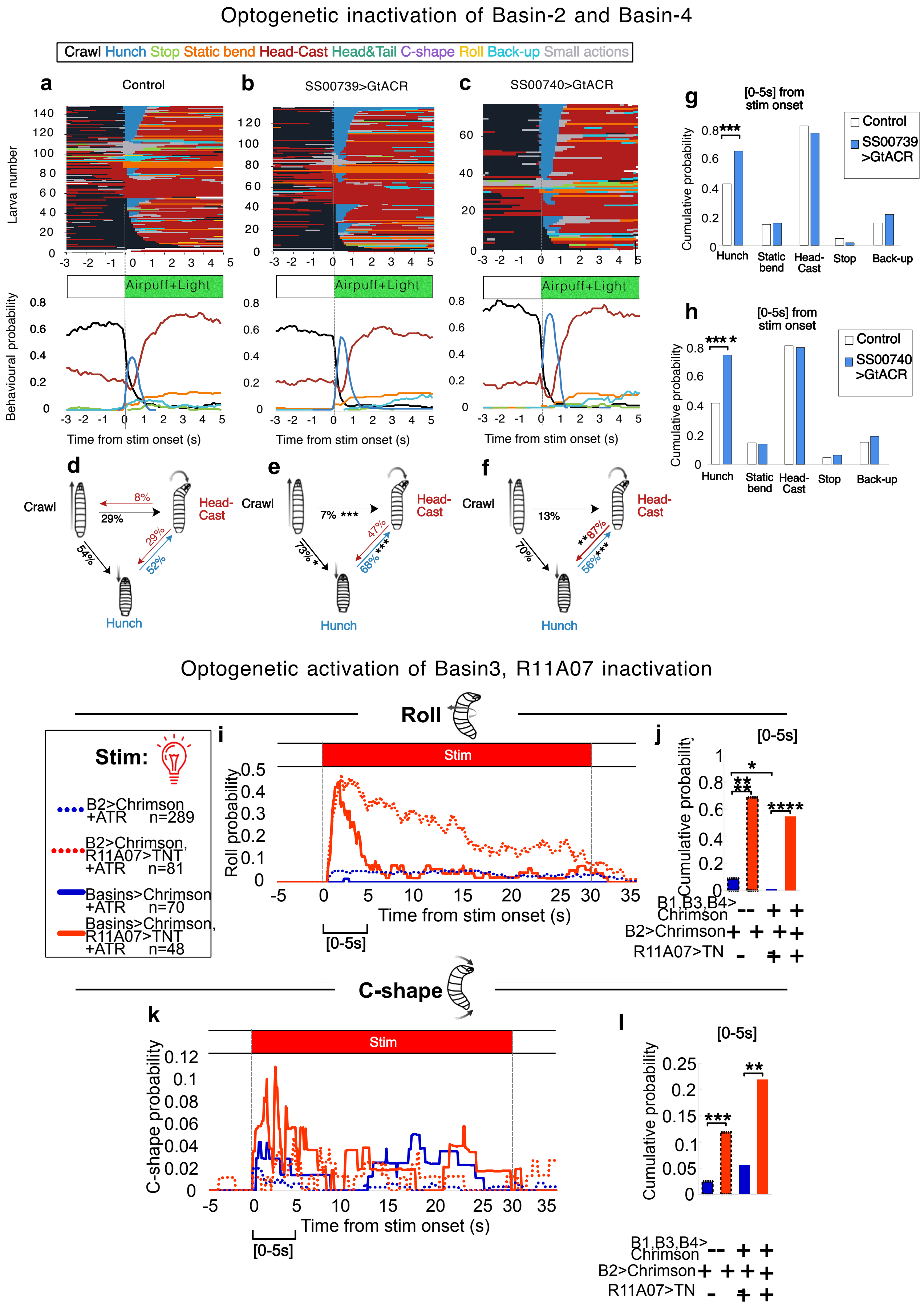

Optogenetic inactivation of Basin-2 and Basin-4
Crawl Hunch Stop Static bend Head-Cast Head&Tail C-shape Roll Back-up Small actions
a
Control
b
SS00739>GtACR
c
SS00740>GtACR
Larva number
Airpuff+Light
Behavioural probability
Time from stim onset (s)
g
[0-5s] from stim onset
Control
SS00739 >GtACR
Cumulative probability
Hunch
Static bend
Head-Cast
Stop
Back-up
h
[0-5s] from stim onset
Control
SS00740 >GtACR
d
e
f
Crawl
Head-Cast
Hunch
Optogenetic activation of Basin3, R11A07 inactivation
Roll
Stim:
B2>Chrimson +ATR n=289
B2>Chrimson, R11A07>TNT +ATR n=81
Basins>Chrimson +ATR n=70
Basins>Chrimson, R11A07>TNT +ATR n=48
i
Stim
Roll probability
Time from stim onset (s)
[0-5s]
j
[0-5s]
Cumulative probability
B1,B3,B4> Chrimson
B2>Chrimson
R11A07>TN
C-shape
k
Stim
C-shape probability
Time from stim onset (s)
[0-5s]
l
[0-5s]
Cumulative probability
B1,B3,B4> Chrimson
B2>Chrimson
R11A07>TN

Supplementary Figure 8. a-f. Optogenetic inactivation of Basin-2 (SS00739) and Basin-4 (SS00740) with GtACR during air puff responses (4 m/s) using green light (0.9 mW/cm2). **a.** control larvae (attP2-40>GtACR, n=145), **b.** larvae with SS00739 neurons optogenetically inactivated (SS00739>GtACR, n=136), **c.** larvae with SS00740 neurons optogenetically inactivated (SS00740>GtACR, n=77). **a-c.** ethogram (top), mean behavioral probabilities over time (bottom). Stim onset at 60 s. **d-f.** Transition probabilities cumulated over the first three seconds after stim. onset. Only transition probabilities of 3% or more are shown **d.** Control, **e.** SS00739>GtACR, **f.** SS00740>GtACR. **g,h.** behavioral probability cumulated over the first five seconds after air puff onset, **g.** Control larvae (white), larvae with SS00739 optogenetically inactivated (light blue). **h.** Control larvae (white), larvae with SS00740 optogenetically inactivated (light blue). **i,j.** Rolling probability in response to optogenetic activation of Basins (light alone): Activation of Basin-2 alone (Blue dashed line, n=289), with R11A07 inactivation (Red dashed line, n= 81). Activation of all Basins (blue full line, n=70), with R11A07 inactivation (Red full line , n=48). **i.** Rolling probability over time **j.** Rolling probability cumulated over the first 5 seconds after stim onset. **k-l.** C-shape probability in response to optogenetic activation of Basins (light alone). Colors and animal numbers as in i-j. Fewer Rolls are observed upon optogenetic activation of Basin-2 and all Basins with light alone compared to when optogenetic activation was presented and simultaneously with air puff stimulation (Fig. 8i-k). A potential explanation for the absence of Rolling upon optogenetic activation of Basins in our hands compared to previous work could be the use of different drivers and/or different effectors (L72F11, L38H09 and Chrimson in this study and TrpA and R72F11 in Ohyama *et al.*, 2015). In addition, the L38H09 (LexA) labels the Basin-2 neurons stochastically in different segments (see below Supplementary Fig. 9) which is likely to have an effect on the phenotype observed (for example the Tail Casts). Light intensity: 0.3 mW/cm², air puff intensity: 4 m/s. *:p<0.05, **:p<0.005, ***:p<0.0005, ****:p<0.0001, Chi² test, two-sided. The source data and p values are provided in Source Data 5.

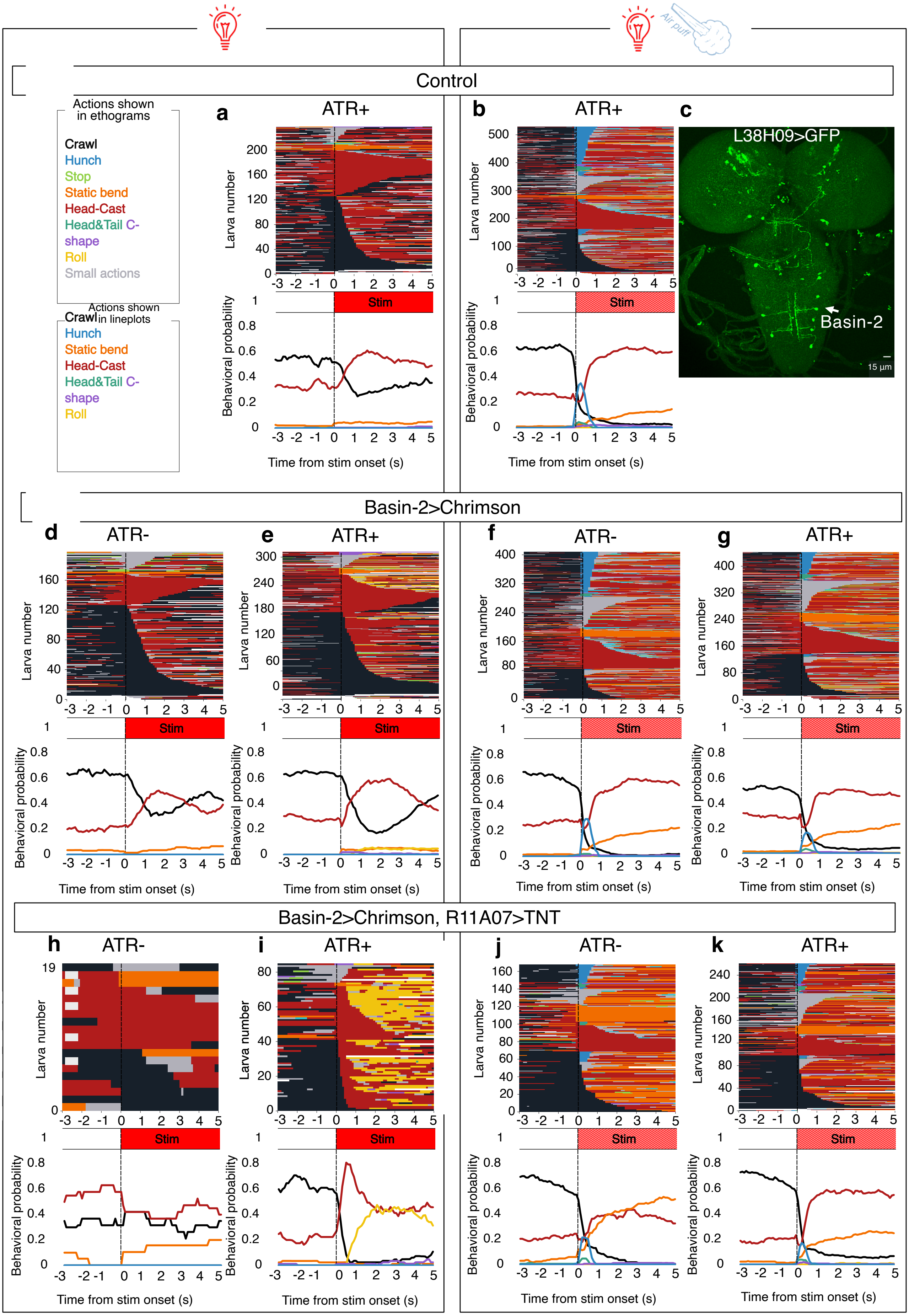

Air puff
Control
Actions shown in ethograms
Crawl
Hunch
Stop
Static bend
Head-Cast
Head&Tail C-shape
Roll
Small actions
Actions shown in lineplots
Crawl
Hunch
Static bend
Head-Cast
Head&Tail C-shape
Roll
a
ATR+
b
ATR+
c
L38H09>GFP
Basin-2
15 µm
Larva number
Behavioral probability
Stim
Time from stim onset (s)
Basin-2>Chrimson
d
ATR-
e
ATR+
f
ATR-
g
ATR+
Basin-2>Chrimson, R11A07>TNT
h
ATR-
i
ATR+
j
ATR-
k
ATR+

Supplementary Figure 9. Impact of R11A07 inactivation on the Basin-2-induced behavioral responses. Ethogram and behavioral probabilities over time showing all observed actions **a-b.** Control (attP2-40>CsChrimson larvae were reared on food medium supplemented with ATR). **a.** light stimulation (n=199). **b.** air puff and light stimulation (n=509). Top: ethogram, showing behavioral sequences of each individual (one line in the ethogram) as a function of time. Bottom: mean behavioral probability across the population. **c.** Expression profile of the L38H09 line. Scale bar: 15 µm **d-g.** Optogenetic activation of Basin-2 (L38H09>CsChrimson) alone (d,e) or combined with air puff stimulation (f,g) **d.** larvae reared without ATR (n=170) **e.** Larvae reared with ATR (n=289) **f.** Larvae reared without ATR (n=395) **g.** Larvae reared with ATR (n=433). **h-k.** Optogenetic activation of Basin-2 (L38H09>CsChrimson) with R11A07 inactivated (R11A07>TNT). Optogenetic activation alone (h,i) or combined with air puff stimulation (j,k) **h.** n=19, **i.** n=81 **j.** n=166 **k.** n=254. Light intensity: 0.3mW/cm², air puff intensity: 4m/s.

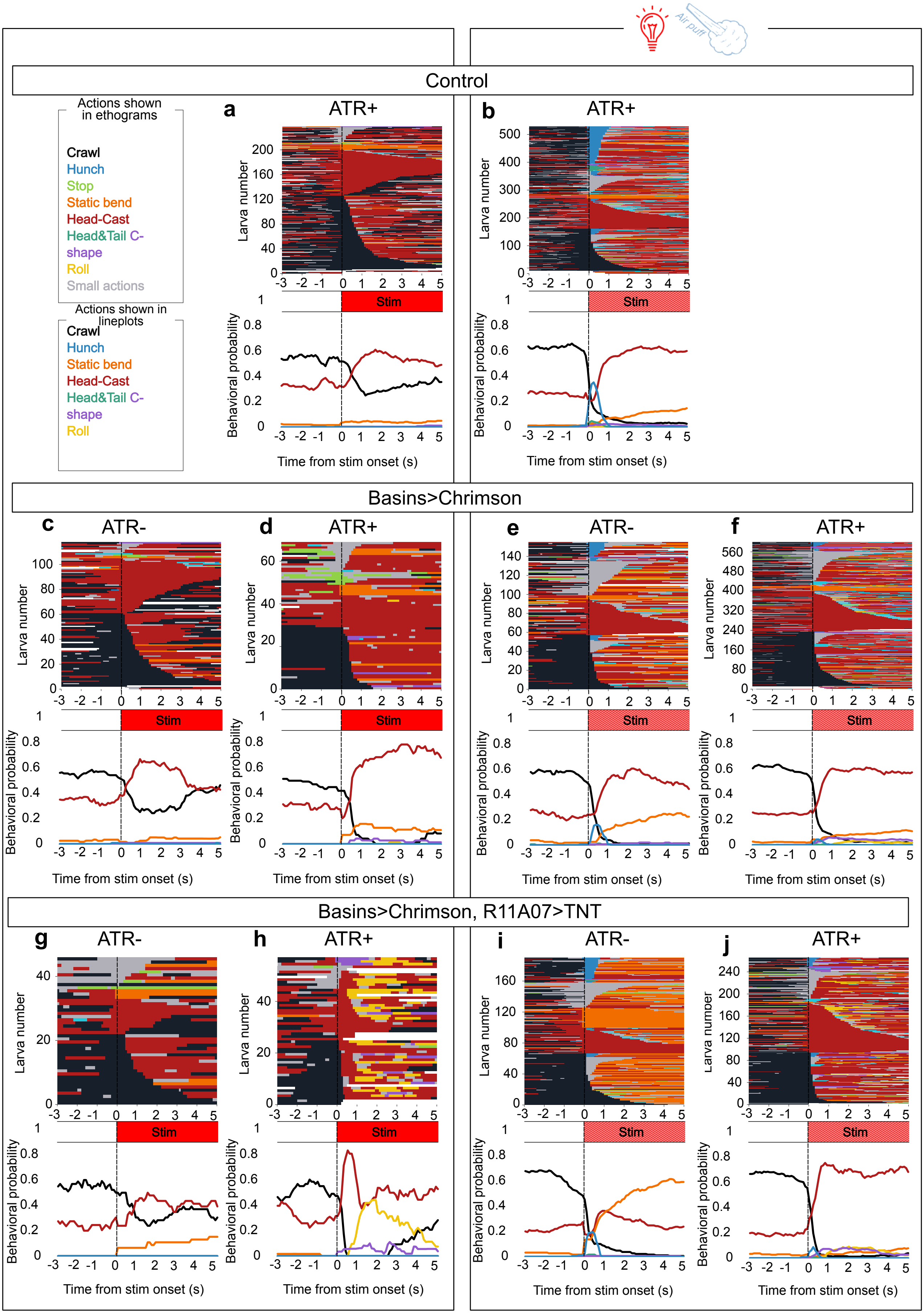
Air puff
Control
Actions shown in ethograms
Crawl
Hunch
Stop
Static bend
Head-Cast
Head&Tail C-shape
Roll
Small actions
Actions shown in lineplots
Crawl
Hunch
Static bend
Head-Cast
Head&Tail C-shape
Roll
a
ATR+
b
ATR+
Larva number
Stim
Behavioral probability
Time from stim onset (s)
Basins>Chrimson
c
ATR-
d
ATR+
e
ATR-
f
ATR+
Basins>Chrimson, R11A07>TNT
g
ATR-
h
ATR+
i
ATR-
j
ATR+

Supplementary Figure 10. Impact of R11A07 inactivation on the all-Basins-induced behavioral responses. Ethogram and behavioral probabilities over time showing all observed actions. **a-b.** Control (attP2-40>CsChrimson, larvae were reared on food medium supplemented with ATR). **a.** light stimulation (n=199) **b.** air puff and light stimulation (n=509). Note, this is the same control as in Supplementary fig. 9a,b. Top: ethogram, showing behavioral sequences of each individual (one line in the ethogram)as a function of time. Bottom: mean behavioral probability across the population. **c-f.** Optogenetic activation of Basins (L72F11>CsChrimson) alone (c,d) or combined with air puff (e,f) **c.** n=102, **d.** n=72, **e.** n=147, **f.** n=583**. g-j.** Optogenetic activation of Basins (L72F11>CsChrimson) with R11A07 inactivated (R11A07>TNT). Optogenetic activation alone (g,h) or combined with air puff (i,j) **g.** n=45 **h.** n=48 **i.** n=190, **j.** n=255. Light intensity: 0.3mW/cm^2, air puff intensity: 4m/s.

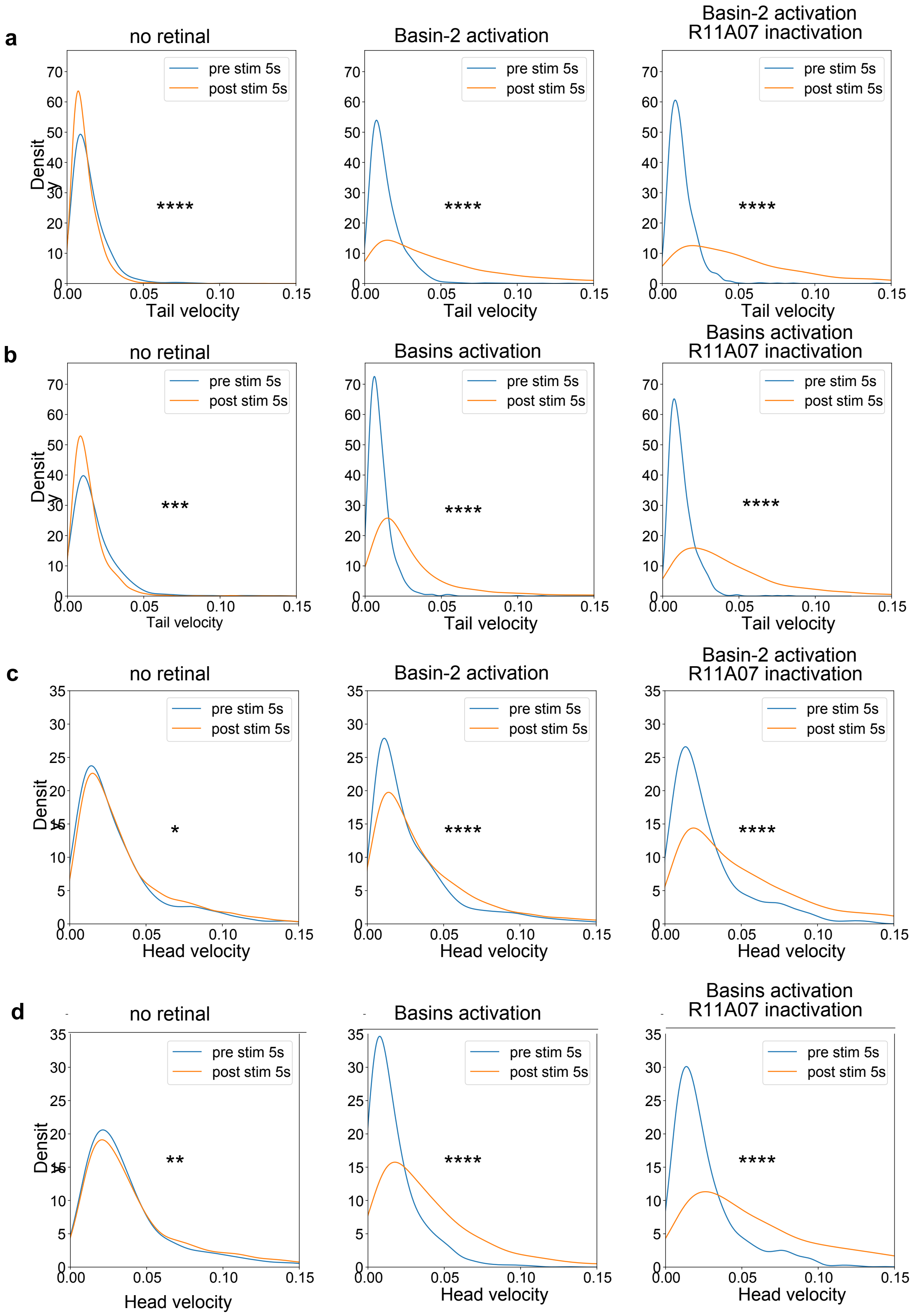

Supplementary Figure 11. Distribution of head and tail velocities upon Basin activation with and without R11A07-labeled neurons a,b. Tail velocity (in s^{-1}) (during Head cast) during the first five seconds upon stimulation compared to a same duration time window prior to stimulus onset. **c,d.** Head velocity (in s^{-1}) (during Head cast) during the first five seconds upon stimulation compared to a same duration time window prior to stimulus onset.) *:p<0.05, **:p<0.001, ***:p<0.0001, ****:p<0.00001, Kolmogorov-Smirnov test, two sided. Genotypes and N as in Figures 9 and 10.

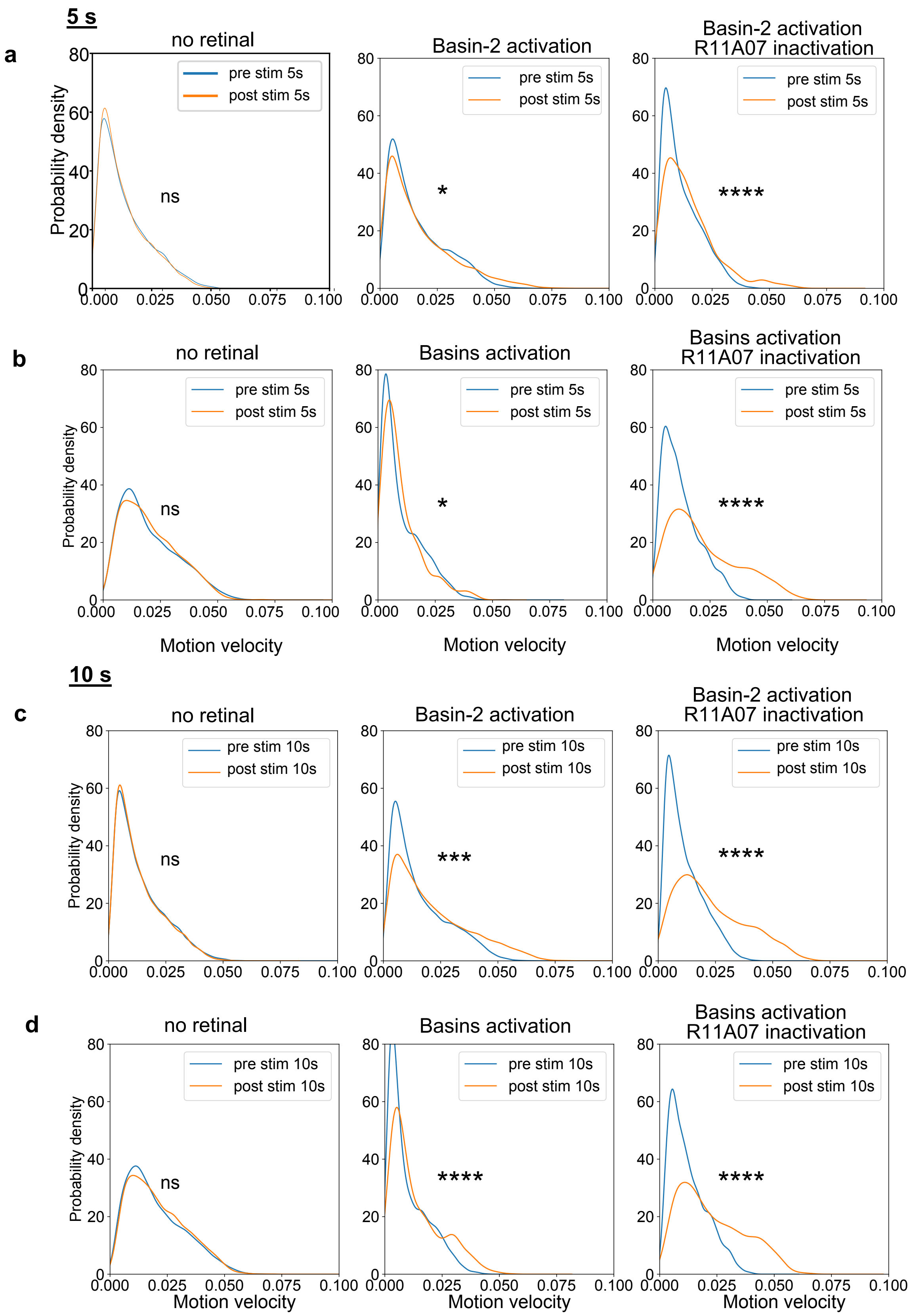

Supplementary Figure 12. Distribution of Motion velocities upon Basin activation with and without R11A07-labeled neurons a,b. Motion velocity during crawls (in s^{-1}) during the first five seconds upon stimulation compared to a same duration time window prior to stimulus onset. **c,d.** Motion velocity during crawls (in s^{-1}) during the first 10 seconds upon stimulation compared to a same duration time window prior to stimulus onset. *:p<0.05, **:p<0.001, ***:p<0.0001, ****:p<0.00001, Kolmogorov-Smirnov test, two sided. Genotypes and N as in Figures 9 and 10.

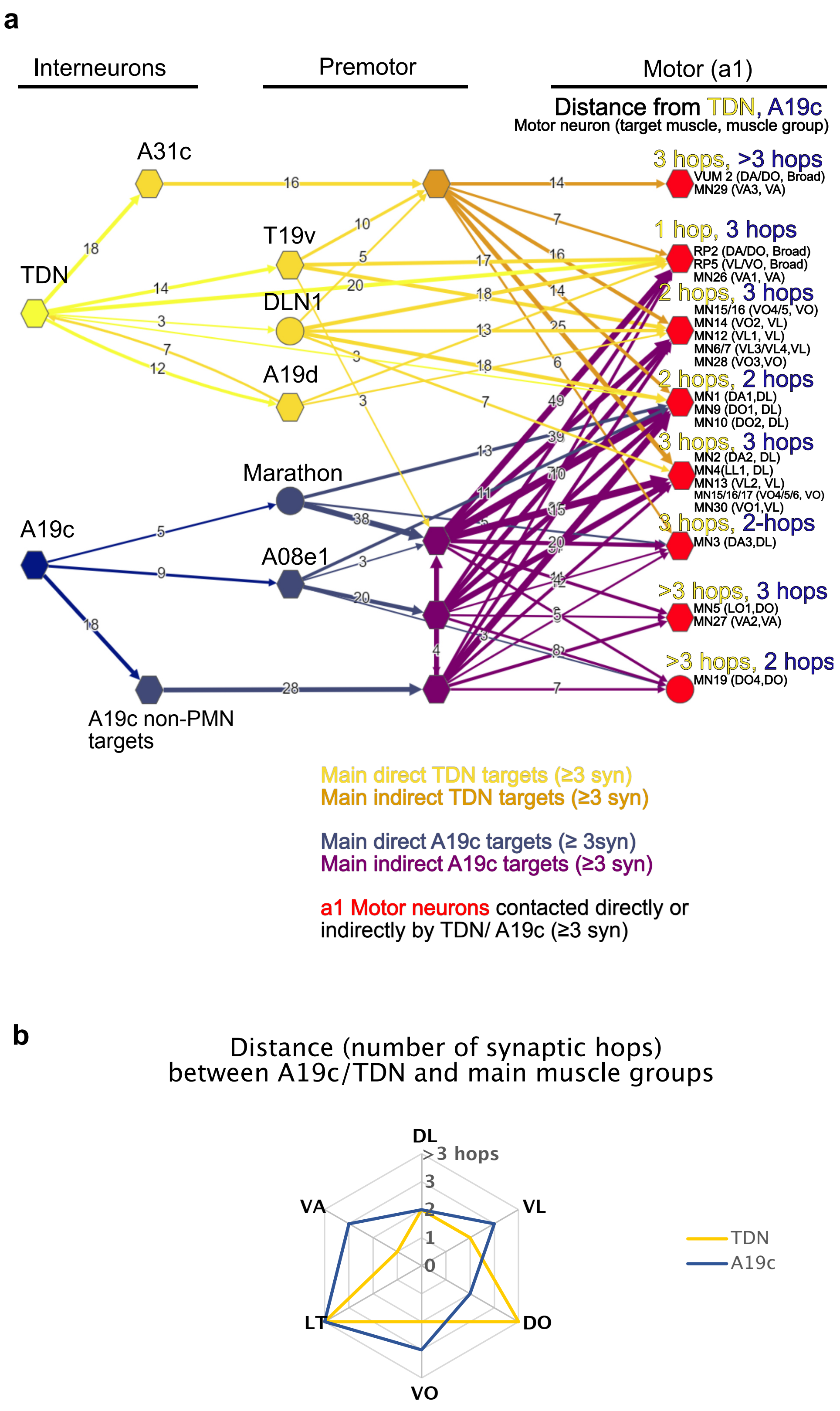

Supplementary Figure 13. Detailed postsynaptic connectivity of TDN, A19c. a. Main pathways from TDN to a1 motor neurons (MNs), and from A19c to a1 MNs. Neurons that are shown receive 3 or more synapses (≥ 3 syn) from either TDN or A19c, that in turn are significantly connected (≥ 3 syn) to either a1 MNs or to premotor neurons (indirect targets). Only the premotor neurons that were significantly connected (≥ 3 syn) to any a1 MNs were taken into account. Neurons which received significant input from TDN/A19c, but did not connect significantly to a1 MNs were excluded. 22 MNs from segment a1 received significant (≥ 3 syn) direct or indirect contact from TDN and/or A19c. These 22 MNs were placed into 8 groups (red), depending on their synaptic distance to TDN and A19c. The full list of neurons shown in this graph is detailed in Source Data 6. **b.** Radar chart summarizing synaptic distance of TDN and A19c from the main muscle groups.

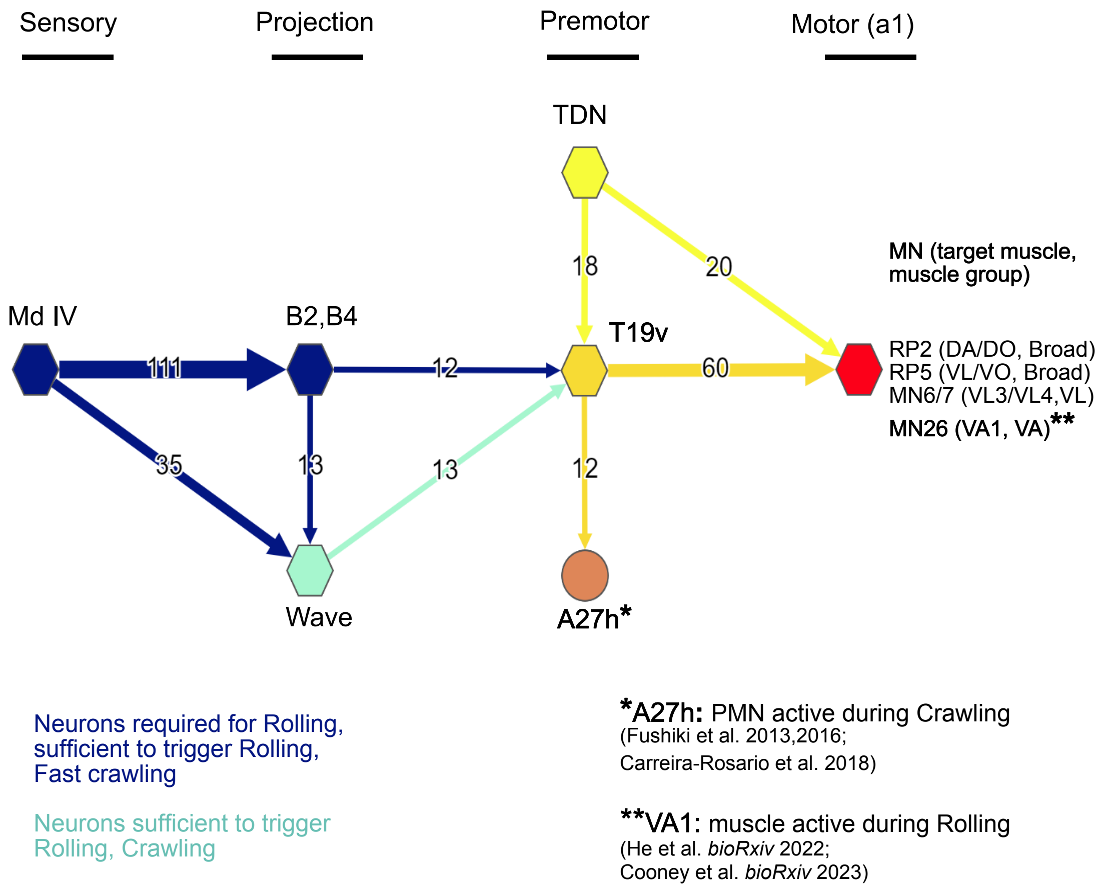

Supplementary Figure 14. premotor neuron T19v is connected to neurons involved in Rolling and Crawling. Neurons shown in the connectivity graph: T19v (t1,t2) and its main presynaptic partners shown to be involved in Rolling: Wave (a2), Basins 2 and 4 (a1), both receiving input from Md IV nociceptive neurons (a1), TDN, which this study suggests may inhibit Rolling. The main motor neurons contacted by T19v are also shown. Among them is motor neurons (MN) MN26, controlling the muscle ventral acute 1 (VA1), recruited during Rolling. The premotor neuron A27h (here from segment a3), shown to be significantly involved in Forward Crawling, is one of the main targets of T19v. Since Rolling is often followed by Fast-Crawling, T19v position makes it an interesting candidate for the sensorimotor control of Rolling but also for the motor control of the transition from a Roll into a Fast-Crawl. It should be noted that T19v also receives significant input from Wave neurons in neuromere a1, but due to the segment-specific nature of Wave connectivity and function, only Wave from neurome a2 was included, as activation of Wave in segments a2-a6 was shown to trigger Rolling (Takagi et al. 2017). This connectivity graph was made with the web-based EM database software CATMAID. The source data are provided in Source Data 6.

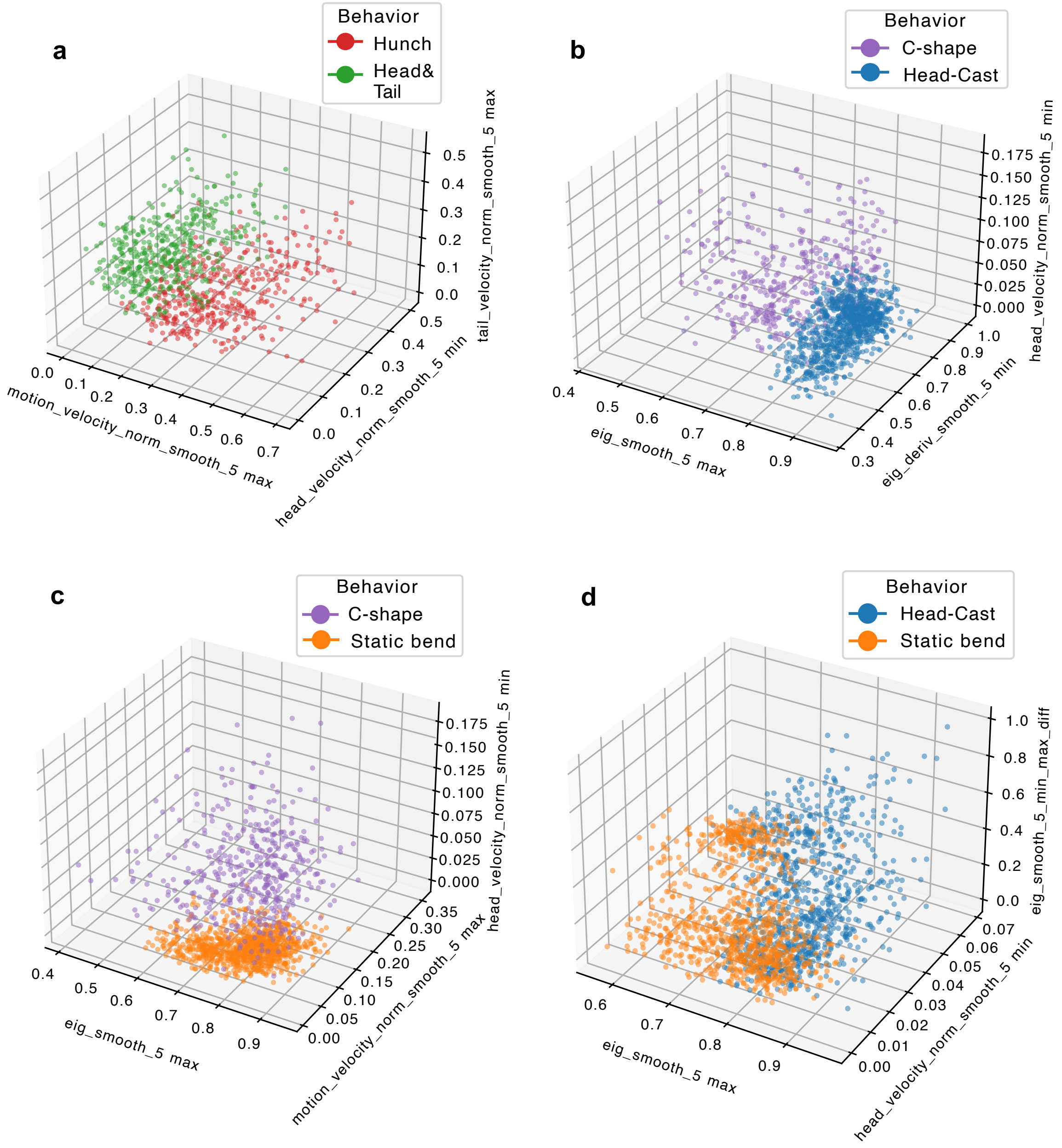

Supplementary Figure 15. 3D scatter plots of selected features used by the classifier show clear separation between the behaviors of interest in the feature space. Each feature was independently normalized and outliers were removed using the interquartile range method. Features were chosen to emphasize the separability of the behaviors. For the behaviors C-shape, Head-Cast, and Static bend, a random subset of the data was plotted with N=2000 samples per behavior. For the behaviors Hunch and Head-Tail, N=483 samples per behavior were plotted. The axes for each behavior pair are as follows. **a.** Hunch and Head-and-Tail : norm of the motion velocity in x-axis, norm of the head velocity in y axis and nomr of the tail velocity in z-axis. **b.** C-shape and Head Cast: λ in the x-axis, dλ.dt in the y-axis and the norm of the head velocity in the z-axis **c.** C-shape and Static bend: λ in the x-axis, nomr of the motion velocity in y-axis, norm of the head velocity in z-axis. **d.** Head Cast and Static bend: λ in the x-axis, norm of the head velocity in y-axis and max(λ) - min(λ) in z-axis.

Supplementary Method 1 Resource Table

	REAGENT or RESOURCE	SOURCE	IDENTIFIER (official)	SHORT NAME
**Experimental Models: Organisms/Strains**	**Fly Stocks**			
Drosophila,	Canton S			CSMH
	y w;;attP2	Pfeiffer et al, 2008, 10	Pfeiffer et al, 2008, 10	attP2
	y w; attP40; attP2	Pfeiffer et al, 2010	Pfeiffer et al, 2010	attP2-40
	w+; UAS-TNTe		Sweeney et al, 1995	UAS-TNT
	20XUAS-CsChrimson-mVenus trafficked in attP18	Bloomington	55134	UAS-Chrimson
	w 1118			w1118
	pGP-JFRC7-20XUAS-IVS-GCaMP6s 15.641 in VK00005	Bloomington	42749	UAS-GCaMP6s
	w[1118]; P{y[+t7.7] w[+mC]=GMR38H09-lexA}attP40	Bloomington	54102	L38H09
	w[1118]; P{y[+t7.7] w[+mC]=13XLexAop2-IVS-GCaMP6s-p10}su(Hw)attP1	Bloomington	44274	LexAop-GCaMP6s
	w[1118]; P{y[+t7.7] w[+mC]=GMR11A07-GAL4}attP2	Bloomington	49826	11A07Gal4
	72F11-LexAp65 in attP40	Bloomington	94661	L72F11
	w1118(5905); +; pJFRC12-10XUAS-IVS-myr::GFP in attP2	Bloomington	32197	UAS-GFP
	w[1118]; P{y[+t7.7] w[+mC]=13XLexAop2-IVS-myr::GFP}su(Hw)attP1	Bloomington	BDSC 32212	LexAop-GFP
	UAS-FLP; 11F02-gal80; EL-Gal4	Ellie Heckscher (UChicago)	Wreden et al, 2017	EL-Gal4
	UAS-FLP; EL-AD; 11F02-DBD	Ellie Heckscher (UChicago)	Heckscher et al., 2015, Wreden et al, 2017	EL-Gal4
	w[1118]; P{y[+t7.7] w[+mC]=GMR20B01-GAL4}attP2	Bloomington	48877	20B01-Gal4
	GMR_SS00739	Jovanic et al, Cell 2016	Jovanic et al, Cell 2016	SS00739-Gal4
	GMR_SS00740	Jovanic et al, Cell 2016	Jovanic et al, Cell 2016	SS00740-Gal4
	w[1118]; P{y[+t7.7] w[+mC]=GMR38H09-GAL4}attP2	Bloomington	50030	38H09-Gal4
	w[1118]; P{y[+t7.7] w[+mC]=GMR57F07-lexA}attP40	Bloomington	54899	57F07-LexA
	w[1118]; P{y[+t7.7] w[+mC]=GMR72F11-GAL4}attP2	Bloomington	39786	
	11A07-LexAp65 in attP40	Bloomington	52427	11A07-LexA
	11A07-LexAp65 in VK00022	Bloomington	2125705	11A07-LexA
	11A07-LexAp65 in VK00002	Bloomington	2125809	11A07-LexA
	w[1118];P{y[+t7.7] w[+mC]=R11A07-p65.AD}attP40	Bloomington	75685	
	w[1118];P{y[+t7.7] w[+mC]=R65E09-p65.AD}attP40;MKRS/TM6B, Tb[1]	Bloomington	70974	
	w[1118];P{y[+t7.7] w[+mC]=R65E09-ZpGDBD in attP2	Bloomington	69431	
	w[1118];P{y[+t7.7] w[+mC]=R11A07-ZpGDBD in attP2	Bloomington	68722	
	20B01-LexAp65 (JK22c); 20xUAS-CsChrimson-mCherry-trafficked in su(Hw)attP1 (3015633), 13xLexAop2-IVS-GCaMP6s-p10 50.641 in VK5	Jovanic et al, Cell 2016	Jovanic et al, Cell 2016	20B01-lexA ; LexAop-GCamp6s, UAS-CsChrimson-mCherry

Supplementary Method 1 Resource Table

	w-; 38H09-LexA (AttP40)/CyO; 20xUAS-CsChrimson-mCherry-trafficked in su(Hw)attP1 (3015633), 13xLexAop2-IVS-GCaMP6s-p10 50.641 in VK5	this paper	this paper	Construction Eloïse
	;LexAopChrimson,UAS-TNT/CyO Tb;11A07Gal4/Tm6 TbSb	this paper	this paper	;LexAopChrimson,UAS-TNT/CyO Tb;11A07Gal4/Tm6 TbSb
	;LexAopChrimson,UAS-TNT/CyO;	this paper	this paper	;LexAopChrimson,UAS-TNT/CyO;
	w[1118];P{y[+t7.7] w[+mC]=R11A07-p65.AD}attP40/CyO,Tb; P{y[+t7.7] w[+mC]=R65E09-ZpGDBD in attP2/TM6,TbSb	this paper	this paper	11A07AD;65E09DBD
	w[1118];P{y[+t7.7] w[+mC]=R65E09-p65.AD}attP40/Sp; 11A07-ZpGDBD in attP2/TM6,TbSb	this paper	this paper	65E09AD;11A07DBD
	w[*]; P{y[+t7.7] w[+mC]=UAS-GtACR1.d.EYFP}attP2	Bloomington	92983	GtACR

	**Chemical, peptides and recombinant proteins**			
	all-trans-retinal	TRC Candada	Cat #: R240000	

**Antibodies**					
Primary antibodies					
species, target	Supplier	Catalog number / clone name	Dilution	Lot number	Antibody validation
chicken anti-GFP	Invitrogen	A10262	1/1000	2156242	the antibody has been validated by detection of different targets fused to GFP tag in transiently transfected lysates tested as described by the supplier (https://www.thermofisher.com/antibody/product/GFP-Antibody-Polyclonal/A10262).
mouse anti-CHAT	DSHB	ChAT4B1-c (concentrate)	1/50	1ea12/6/18	the antibody has been validated as described by Salvaterra (Neuroscience research, 1996).
rabbit anti-GABA	SIGMA	A2052	1/500	029M4830V	expression of GABA was detected in neucortical cells from the brains of E19 day old rat embyros as described by the supplier (https://www.sigmaaldrich.com/FR/fr/product/sigma/a2052).
rabbit anti-DVGLUT	gift from Hermann Aberle		1/500		the antibody has been validated as described by Mahr and Aberle (Gene Expr. Patterns, 2006).
rabbit anti-DsRed	Clontech	632496	1/500	1509043	the quality and performance of the antibody was tested by Western blot analysis as described by the supplier (https://www.takarabio.com/documents/Certificate%20of%20Analysis/632496/632496-101717.pdf?srsltid=AfmBOooVitSlvN_fdUhMDUNSwM6xA0yTDRrTR5Egbaomb9X5qJcIZrp7)
Secondary antibodies					
species, target, coupled-fluorophore	Supplier	Catalog number	Dilution	Lot number	

Supplementary Method 1 Resource Table

goat anti-chicken AlexA Fluor 488	Abcam	ab150169	1/1000	GR3234906-2	
goat anti-mouse Alexa Fluor 647	Jackson Immuno Research	115-605-003	1/200	145779	
goat anti-rabbit Alexa Fluor 647	Jackson Immuno Research	111-605-003	1/200	146340	
goat anti-rabbit Cy3	Jackson Immuno Research	111-165-144	1/500	163657	
**Softwares and algorithms**					
**Name**	**Source**	**Identifier**			
CATMAID	http://www.catmaid.org	Saalfeld et al., 2009,			
MATLAB	http://www.mathworks.org				
MWT	http://sourceforge.net/projects/mwt	Swierczek et al., 2011			
