# Supplementary material for: Neural circuits underlying context-dependent competition between defensive actions in Drosophila larvae
Source: Nat Commun. 2025 Jan 28;16:1120. doi: 10.1038/s41467-025-56185-2 (PMC11775277; doi:10.1038/s41467-025-56185-2)
Supplement: Supplementary file 3 — Description of Additional Supplementary Files [file 41467_2025_56185_MOESM3_ESM.pdf]

Description of Additional Supplementary Files

Supplementary Movie 1. Example of Hunching, C-Shape and Roll from SPARC R11A07 experiments.
